# A Mathematical Model with Pulse Effect for Three Populations of the Giant Panda and Two Kinds of Bamboo

**DOI:** 10.1155/2013/137384

**Published:** 2013-12-05

**Authors:** Xiang-yun Shi, Guo-hua Song

**Affiliations:** ^1^College of Forestry, Beijing Forestry University, Beijing 100083, China; ^2^College of Mathematics and Information Science, Xinyang Normal University, Xinyang, Henan, 464000, China; ^3^School of Science, Beijing University of Civil Engineering and Architecture, Beijing 100044, China

## Abstract

A mathematical model for the relationship between the populations of giant pandas and two kinds of bamboo is established. We use the impulsive perturbations to take into account the effect of a sudden collapse of bamboo as a food source. We show that this system is uniformly bounded. Using the Floquet theory and comparison techniques of impulsive equations, we find conditions for the local and global stabilities of the giant panda-free periodic solution. Moreover, we obtain sufficient conditions for the system to be permanent. The results provide a theoretical basis for giant panda habitat protection.

## 1. Introduction

The giant panda is a highly specialized Ursid, approximately its 99% of their diet is bamboo [1]. Many of these bamboo species sexually reproduce by synchronous semelparity, that is, the bamboos of a given species within a given region flower at the same time and then die. If the particular bamboo species is one that pandas locally depend upon, there can be a great reduction in local carrying capacity. For example, in the middle of the 1970s and the beginning of 1980s, a large area of Fargesia denudata in Minshan Mountains and Bashania fangiana in Qionglai Mountains bloomed and died, causing the death of at least 138 and 144 giant pandas, respectively [2].

Yuan et al. may be the first person who have developed mathematical models for the relationship between giant pandas and bamboo [3]. After that some mathematical models are presented by some scholars [4, 5]. Guo et al. described an improved mathematical model for the relationship between the populations of giant pandas (*Ailuropoda melanoleuca*) and bamboo by adding a correction term which takes into account the effect of a sudden collapse of bamboo as a food source [5]. Modified by the above, we shall establish an ecological model of the population ecology on the three populations of the giant panda and two kinds of bamboo.

Impulsive differential equations, that is, differential equations involving an impulse effect, appear as a natural description of observed evolution phenomena of several real-world problems [6, 7]. It is known that many biological phenomena involving thresholds, bursting rhythm models in biology, do exhibit impulse effects. The differing varieties of bamboo go through periodic die-offs as part of their renewal cycle. The bamboo, at the end of its life cycle, will bloom and drop its seeds and then dies. Often vast areas of the bamboo forest disappear at the same time. Generally died-back bamboo should take from 10 to 20 years before it can support a panda population again [1, 8]. So we can use impulse effect to describe bamboo flowering phenomenon. In this paper, we will consider an impulsive differential system of the population ecology on the three populations of the giant panda and two kinds of bamboo:

(1) $$\begin{array}{c} \frac{dx_{1}}{dt}=x_{1}\left(a_{10}-a_{11}x_{1}-a_{13}x_{3}\right),\;t\neq \left(n+l-1\right)T,\\ \frac{dx_{2}}{dt}=x_{2}\left(a_{20}-a_{22}x_{2}-a_{23}x_{3}\right),\;t\neq nT,\\ \frac{dx_{3}}{dt}=x_{3}\left(-a_{30}-a_{33}x_{3}+a_{31}x_{1}+a_{32}x_{2}\right),\\ \Delta x_{1}\left(t\right)=-\alpha x_{1}\left(t\right),\;t=\left(n+l-1\right)T,\\ \Delta x_{2}\left(t\right)=-\beta x_{2}\left(t\right),\;t=nT,\\ \left(x_{1}\left(0^{+}\right),x_{2}\left(0^{+}\right),x_{3}\left(0^{+}\right)\right)=\left(x_{10},x_{20},x_{30}\right), \end{array}$$

where *x*_1_(*t*) and *x*_2_(*t*) are the respective densities of two kinds of bamboo at time *t* and *x*_3_(*t*) is the density of the giant panda. *a*_*i*0_  (*i* = 1,2) denote the birthrate of two kinds of bamboo, respectively. *a*_*ii*_  (*i* = 1,2) denote the density restriction coefficients of the two kinds of bamboo. *a*_*i*3_  (*i* = 1,2) are the predation rate of giant panda feeding upon two kinds of bamboo, respectively. (*a*_3*i*_/*a*_*i*3_)  (*i* = 1,2) are the transformation rate of giant panda due to predation on bamboo. Most predator-prey relationships are complicated by the predator's use of multiple prey items or by prey being used by multiple predators. The bamboo-panda relationship does, however, simplify to a binary one such as those modelled by the Lotka-Volterra equations. Although giant pandas do eat other items, their limited remaining habitat has reduced their ability to move on to other species of bamboo which are not flowering [9–11].

The organization of the paper is as follows.  Section 2 deals with some notation and definitions together with a few auxiliary results related to the comparison theorem, positivity, and boundedness of solutions.  Section 3 is devoted to studying the stability of the giant panda-free periodic solutions. In Section 4, we find the conditions which ensure the giant panda to be permanent. The paper ends with discussion on the results obtained in the previous sections.

## 2. Preliminaries

In this section we will introduce some notations and definitions together with a few auxiliary results related to the comparison theorem, which will be useful for establishing our results.

Let ℝ_+_ = [0, +*∞*), ℝ_+_* = (0, +*∞*), and ℝ_+_^3^ = {*x* = (*x*_1_, *x*_2_, *x*_3_) ∈ ℝ^3^ : *x*_1_, *x*_2_, *x*_3_ ≥ 0}. Denote as *ℕ* the set of all of nonnegative integers and as *f* = (*f*_1_, *f*_2_, *f*_3_)^*T*^ the right-hand sides of the first three equations (1). Let *V* : ℝ_+_ × ℝ_+_^3^ → ℝ_+_, and then *V* is said to belong to class *V*_0_ if

1. *V*  is continuous on ((*n* − 1)*T*, (*n* + *l* − 1)*T*] × ℝ_+_^3^ ∪ ((*n* + *l* − 1)*T*, *nT*] × ℝ_+_^3^ and lim⁡_(*t*,*y*)→(*t*_0_,*x*)_⁡*V*(*t*, *y*) = *V*(*t*_0_, *x*) exists, where *t*_0_ = (*n* + *l* − 1)*T* ^+^ and *nT* ^+^.
2. *V* is locally Lipschitzian in *x*.

**Definition 1**

Let *V* ∈ *V*_0_, for (*t*, *x*)∈((*n* − 1)*T*, (*n* + *l* − 1)*T*] × ℝ_+_^3^, and the upper right derivative of *V* with respect to the impulsive differential system (1) is defined as

(2) $$\begin{array}{c} D^{+}V\left(t,x\right)=\underset{h\to 0_{+}}{\mathrm{limsup}}\frac{1}{h}\left[V\left(t+h,x+hf\left(t,x\right)-V\left(t,x\right)\right)\right]. \end{array}$$

The solution of system (1) is piecewise continuous function *X* : ℝ_+_ × ℝ_+_^3^, *X*(*t*) is continuous on ((*n* − 1)*T*, (*n* + *l* − 1)*T*]∪((*n* + *l* − 1)*T*, *nT*] and *X*(*t*_0_^+^) = lim⁡_*t*→*t*_0__⁡*X*(*t*) exists, where *t*_0_ = (*n* + *l* − 1)*T* ^+^ and *nT* ^+^. The smoothness properties of *f* guarantee the global existence and uniqueness of solution of system (1) for details, see [12]. Given a solution *X*(*t*) of (1), defined on [*t*_0_, *t*_0_ + *a*) with *a* > 0, we say that a solution *Y*(*t*) of (1) is a proper continuation to the right of *X*(*t*) if *Y*(*t*) is defined on [*t*_0_, *t*_0_ + *b*) for some *b* > *a* and *X*(*t*) = *Y*(*t*) for *t* ∈ [*t*_0_, *t*_0_ + *a*). The interval [*t*_0_, *t*_0_ + *a*) is called the maximal interval of existence of solution (saturated solution) *X*(*t*) of (1) if *x*(*t*) is well defined on [*t*_0_, *t*_0_ + *a*) and it doesnot have any proper continuation to the right. For other results on impulsive differential equations, see [12, 13].

**Lemma 2 (see [12])**

Suppose *V* ∈ *V*_0_ and

(3) $$\begin{array}{c} D^{+}V\left(t,x\right)\leq g\left(t,V\left(t,x\right)\right),\;t\neq \left(n+l-1\right)T,\;\;t\neq nT,\\ V\left(t,x\left(t^{+}\right)\right)\leq \varphi_{1}\left(V\left(t,x\right)\right),\;t=\left(n+l-1\right)T,\\ V\left(t,x\left(t^{+}\right)\right)\leq \varphi_{2}\left(V\left(t,x\right)\right),\;t=nT, \end{array}$$

where *g* : ℝ_+_ × ℝ_+_ → ℝ satisfies.

(H):  *g* is continuous on ((*n* − 1)*T*, (*n* + *l* − 1)*T*] × ℝ_+_ ∪ ((*n* + *l* − 1)*T*, *nT*] × ℝ_+_ and the limit lim⁡_(*t*,*y*)→(*t*_0_,*x*)_⁡*g*(*t*, *y*) = *g*(*t*, *x*) exists, where *t*_0_ = (*n* + *l* − 1)*T* ^+^ and *nT* ^+^, and is finite for *x* ∈ ℝ_+_ and *n* ∈ *ℕ*, and *ψ*_1_, *ψ*_2_ : ℝ_+_ → ℝ_+_ are nondecreasing for all *n* ∈ *ℕ*. Let *r*(*t*) be the maximal solution for the impulsive Cauchy problem:

(4) $$\begin{array}{c} \frac{du}{dt}=g\left(t,u\left(t\right)\right),\;t\neq \left(n+l-1\right)T,\;\;t\neq nT,\\ u\left(t^{+}\right)=\psi_{1}\left(u\left(t\right)\right),\;t=\left(n+l-1\right)T,\\ u\left(t^{+}\right)=\psi_{2}\left(u\left(t\right)\right),\;t=nT,\\ u\left(0^{+}\right)=u_{0}\geq 0, \end{array}$$

defined on [0, *∞*). Then *V*(0^+^, *x*_0_) ≤ *u*_0_ implies that *V*(*t*, *x*(*t*)) ≤ *r*(*t*), *t* ≥ 0, where *x*(*t*) is any solution of (3).

Note that under appropriate conditions (such that, *g* is locally Lipschitz continuous with *x* in ((*n* − 1)*T*, (*n* + *l* − 1)*T*] × ℝ_+_ ∪ ((*n* + *l* − 1)*T*, *nT*] × ℝ_+_ etc. see Remark 2.3 and Theorem 2.3 of [13] for the details) the Cauchy problem (3) has a unique solution and in that case *r*(*t*) becomes the unique solution of (4). We now indicate a result which provides estimation for the solution of a system of differential inequalities. Let PC(ℝ_+_, ℝ)(PC^1^(ℝ_+_, ℝ)) denote the class if real piecewise continuous (real piecewise continuously differentiable) functions are defined on ℝ_+_.

**Lemma 3 (see [12])**

Let the function *u* ∈ *PC* ^1^(ℝ_+_, ℝ) satisfy the inequalities:

(5) $$\begin{array}{c} \frac{du}{dt}\leq \left(\geq \right)p\left(t\right)u\left(t\right)+f\left(t\right),\;t\neq \tau_{k},\;\;t>0,\\ u\left(\tau_{k}\right)\leq \left(\geq \right)d_{k}u\left(\tau_{k}\right)+h_{k},\;k\geq 0,\\ u\left(0^{+}\right)\leq \left(\geq \right)u_{0}, \end{array}$$

where *p*, *f* ∈ *PC*(ℝ_+_, ℝ) and *d*_*k*_ ≥ 0, *h*_*k*_ and *u*_0_ are constants, and {*τ*_*k*_}_*k*≥0_ is a strictly increasing sequence of positive real number. Then, for *t* > 0

(6) u(t)≤(≥)u(0)∏0<tk<tdkexp⁡(∫0tp(s)ds) +∑0<tk<t(∏tk<tj<tdjexp⁡(∫tktp(s)ds)hk) +∫0t∏0<tk<tdkexp⁡(∫stp(τ)dτ)f(s)ds.

Using Lemma 3, it is possible to prove that the solution of the Cauchy problem (1) with strictly positive initial value remains strictly positive.

**Lemma 4**

The positive octant (ℝ_+_^3^) is an invariant region for system (1).

**Proof**

Let us consider (*x*_1_(*t*), *x*_2_(*t*), *x*_3_(*t*)):[0, *T*_0_] → ℝ^3^ a saturated solution of system (1) with a strictly positive initial value (*x*_10_, *x*_20_, *x*_30_). By Lemma 2, we can obtain that, for 0 < *t* < *T*_0_,

(7) x1(t)≥x10(1−α)[(t+(1−l)T)/T] ×exp⁡(∫0t[a11x1(s)−a13x3(s)]ds),x2(t)≥x20(1−β)[t/T] ×exp⁡(∫0t[−a22x2(s)−a23x3(s)]ds),x3(t)=x30exp⁡(∫0t(−a30−a33x3+a31x1+a32x2)ds),

where [*x*] represents the largest integer not exceeding *x*. That is, (*x*_1_(*t*), *x*_2_(*t*), *x*_3_(*t*)) remains strictly positive on [0, *T*_0_].

**Lemma 5**

All solutions (*x*_1_(*t*), *x*_2_(*t*), *x*_3_(*t*)) of (1) with initial value (*x*_10_, *x*_20_, *x*_30_) ∈ ℝ_+_^3^ are bounded.

**Proof**

Let (*x*_1_(*t*), *x*_2_(*t*), *x*_3_(*t*)) be a solution of (1) with a positive initial value (*x*_10_, *x*_20_, *x*_30_) and let

(8) $$\begin{array}{c} V\left(t\right)=\frac{a_{31}}{a_{13}}x\left(t\right)+\frac{a_{32}}{a_{23}}x_{2}\left(t\right)+x_{3}\left(t\right),\;t\geq 0. \end{array}$$

Then, if *t* ≠ *nT*, *t* ≠ (*n* + *l* − 1)*T*, and *t* > 0, we obtain that

(9) dVdt=a31a13x1(a10−a11x1)+a32a23x2(a20−a22x2) −a30x3−a33x32.

Then

(10) dVdt+a30V(t)=x1(a10a31a13+a30−a31a11a13x1) +x2(a20a32a23+a30−a32a22a23x2)−a33x32,t≠(n+l−1)T, t≠nT, t>0.

As the right-hand side of (10) is bounded from above denoted by *D*, it follows that

(11) $$\begin{array}{c} \frac{dV}{dt}+a_{30}V\left(t\right)\leq D,\;t\neq \left(n+l-1\right)T,\;\;t\neq nT,\;\;t>0 \end{array}$$

together with

(12) $$\begin{array}{c} V\left(\left(n+l-1\right)T^{+}\right)\leq \left(1-\alpha \right)V\left(\left(n+l-1\right)T\right),\\ V\left(nT^{+}\right)\leq \left(1-\beta \right)V\left(nT\right). \end{array}$$

By Lemma 3, it follows that

(13) V(t)≤V(0+)(∏0<(n+l−1)T<t(1−α)∏0<nT<t(1−β)e−a30t) +D∫0t[∏0<(n+l−1)T<t(1−α)∏0<nT<t(1−β)]e−a30(t−s)ds,

which yields

(14) $$\begin{array}{c} V\left(t\right)\leq V\left(0^{+}\right)e^{-a_{30}t}+\frac{D\left(1-e^{-a_{30}t}\right)}{a_{30}},\;t>0 \end{array}$$

and since the limit of the right-hand side of (14) for *t* → *∞* is

(15) $$\begin{array}{c} V\left(t\right)\leq \frac{D}{a_{30}}<\infty , \end{array}$$

it easily follows that *V*(*t*) is bounded in its domain. Consequently, (*x*_1_(*t*), *x*_2_(*t*), *x*_3_(*t*)) are bounded by a constant “*D*/*a*_30_” for sufficiently lager *t*.

## 3. Stability of the Giant Panda-Free Periodic Solutions

First, we will give the basic properties of the following differential equations considering the absence of the giant panda.

When the giant panda *x*_3_(*t*) is eradicated, it is easy to see that the equations in (1) decouple, and then we consider the properties of the subsystems:

(16) $$\begin{array}{c} \frac{dx_{1}}{dt}=x_{1}\left(a_{10}-a_{11}x_{1}\right),\;t\neq \left(n+l-1\right)T,\\ \Delta x_{1}\left(t\right)=-\alpha x_{1}\left(t\right),\;t=\left(n+l-1\right)T,\\ x_{1}\left(0^{+}\right)=x_{10}, \end{array}$$

(17) $$\begin{array}{c} \frac{dx_{2}}{dt}=x_{2}\left(a_{20}-a_{22}x_{2}\right),\;t\neq nT,\\ \Delta x_{2}\left(t\right)=-\beta x_{2}\left(t\right),\;t=nT,\\ x_{2}\left(0^{+}\right)=x_{20}. \end{array}$$

**Lemma 6 (see [14])**

Suppose that ln⁡(1 − *α*) + *a*_10_ *T* > 0, then the system (16) has a periodic solution *x*_1_*(*t*) with this notation, and the following properties are satisfied:

(18) (i) ∫0Tx1∗(t)dt=1a11[ln⁡⁡(1−α)+a10T],(ii) lim⁡t→∞|x1(t)−x1∗(t)|=0

for all solutions *x*_1_(*t*) of (16) starting with strictly positive *x*_10_.

Similarly, we have the following Lemma 7.

**Lemma 7**

Suppose that ln⁡(1 − *β*) + *a*_20_ *T* > 0, then the system (17) has a periodic solution *x*_2_*(*t*) with this notation, and the following properties are satisfied:

(19) (iii) ∫0Tx1∗(t)dt=1a22[ln⁡⁡(1−β)+a20T],(iv) lim⁡t→∞|x2(t)−x2∗(t)|=0

for all solutions *x*_2_(*t*) of (17) starting with strictly positive *x*_20_.

It follows from Lemmas 6 and 7 that the system (1) has a giant panda-free periodic solution (*x*_1_*(*t*), *x*_2_*(*t*), 0).

Now, we study the local stability of the giant panda-free periodic solution (*x*_1_*(*t*), *x*_2_*(*t*), 0) by means of the Floquent theory. (We can see the details from Page 26 to 35 of [13].)

**Theorem 8**

Suppose that ln⁡(1 − *α*) + *a*_10_ *T* > 0 and ln⁡(1 − *β*) + *a*_20_ *T* > 0 and

(20) −a30T+a31a11(ln⁡(1−α)+a10T)+a32a22(ln⁡(1−β)+a20T)  <0

hold, and then the giant panda-free periodic solution (*x*_1_*(*t*), *x*_2_*(*t*), 0) is locally stable.

**Proof**

The local stability of the periodic solution (*x*_1_*(*t*), *x*_2_*(*t*), 0) may be determined by considering the behavior of small-amplitude perturbations of the solution. Define

(21) $$\begin{array}{c} x_{1}\left(t\right)=u\left(t\right)+x_{1}^{*},\;\;x_{2}\left(t\right)=v\left(t\right)+x_{2}^{*},\\ x_{3}\left(t\right)=w\left(t\right). \end{array}$$

Substituting (21) into system (1), it is possible to obtain a linearization of the system as follows:

(22) du(t)dt=(a10−2a11x1∗(t))u(t)−a13x1∗(t)w(t),t≠(n+l−1)T,dv(t)dt=(a20−2a22x2∗(t))v(t)−a23x2∗(t)w(t),t≠nT,dw(t)dt=(−a30+a31x1∗(t)+a32x2∗(t))w(t),

which can be written as

(23) $$\begin{array}{c} \left(\begin{array}{c} u\left(t\right)\\ v\left(t\right)\\ w\left(t\right) \end{array}\right)=\Phi \left(t\right)\left(\begin{array}{c} u\left(0\right)\\ v\left(0\right)\\ w\left(0\right) \end{array}\right),\;0\leq t\leq T, \end{array}$$

where Φ(*t*) satisfies

(24) dΦ(t)dt =(a10−2a11x1∗0−a13x1∗0a20−2a22x2∗−a23x2∗00−a30+a31x1∗+a32x2∗),

Φ(0) = *I*, and *I* is the identity matrix, so the fundamental solution matrix is

(25) $$\begin{array}{c} \Phi \left(t\right)=\left(\begin{array}{ccc} \exp\left(\int_{0}^{t}\left(a_{10}-2a_{11}x_{1}^{*}\left(s\right)\right)ds\right) & 0 & *\\ 0 & \exp\left(\int_{0}^{t}\left(a_{20}-2a_{22}x_{1}^{*}\left(s\right)\right)ds\right) & **\\ 0 & 0 & \Delta \end{array}\right), \end{array}$$

For the upper triangular matrix, there is no need to calculate the exact forms of ∗ and ∗∗ as it is not required in the analysis that follows. And Δ = exp⁡(∫_0_^*t*^(−*a*_30_ + *a*_31_ *x*_1_*(*s*) + *a*_32_ *x*_2_*(*s*))*ds*).

The resetting impulsive condition of system (1) becomes

(3) Δu(t)=−αu(t),Δv(t)=0,Δw(t)=0, t=(n+l−1)T,Δu(t)=0,Δv(t)=−βv(t),Δw(t)=0. t=nT.

All of the eigenvalues of

(27) $$\begin{array}{c} S=\left(\begin{array}{ccc} 1-\alpha & 0 & 0\\ 0 & 1 & 0\\ 0 & 0 & 1 \end{array}\right)\left(\begin{array}{ccc} 1 & 0 & 0\\ 0 & 1-\beta & 0\\ 0 & 0 & 1 \end{array}\right)\times \left(\begin{array}{ccc} \exp\left(\int_{0}^{t}\left(a_{10}-2a_{11}x_{1}^{*}\left(s\right)\right)ds\right) & 0 & *\\ 0 & \exp\left(\int_{0}^{t}\left(a_{20}-2a_{22}x_{1}^{*}\left(s\right)\right)ds\right) & **\\ 0 & 0 & \Delta \end{array}\right) \end{array}$$

are

(28) λ1=(1−α)exp⁡(∫0t(a10−2a11x1∗(s))ds)=(1−α)exp⁡[−ln⁡(1−α)−a10T],λ2=(1−β)exp⁡(∫0t(a20−2a22x2∗(s))ds)=(1−β)exp⁡[−ln⁡(1−β)−a20T],λ3=exp⁡(∫0t(−a30+a31x1∗(s)+a32x2∗(s))ds)=exp⁡(−a30T+a31a11(ln⁡(1−α)+a10T)    +a32a22(ln⁡(1−β)+a20T)).

Since ln⁡(1 − *α*) + *a*_10_ *T* > 0, ln⁡(1 − *β*) + *a*_20_ *T* > 0, and condition (20) hold, it is obvious that 0 < *λ*_1_ < 1, 0 < *λ*_2_ < 1, and 0 < *λ*_3_ < 1, which implies that (*x*_1_*(*t*), *x*_2_*(*t*), 0) is stable.

If the reverse of (20) is satisfied, then *λ*_3_ > 1 and (*x*_1_*(*t*), *x*_2_*(*t*), 0) is unstable.

**Theorem 9**

If the conditions of Theorem 8 are satisfied, the giant panda-free periodic solution (*x*_1_*(*t*), *x*_2_*(*t*), 0) is globally asymptotically stable.

**Proof**

Choose *ɛ*_1_ > 0, *ɛ*_2_ > 0 small enough that if condition (20) holds,

(29) ρ=exp⁡(−a30T+a31ɛ1T+a32ɛ2T+a31a11[ln⁡⁡(1−α)+a10T]+a32a22[ln⁡(1−β)+a20T])<1.

It is seen that

(30) $$\begin{array}{c} \frac{dx_{1}}{dt}\leq x_{1}\left(a_{10}-a_{11}x_{1}\right),\;t\neq \left(n+l-1\right)T,\\ \Delta x_{1}\left(t\right)=-\alpha x_{1}\left(t\right),\;t=\left(n+l-1\right)T,\\ x_{1}\left(0^{+}\right)=x_{10}, \end{array}$$

and so by Lemmas 2 and 3,

(31) $$\begin{array}{c} x_{1}\left(t\right)\leq x_{1}^{*}\left(t\right)+{ɛ}_{1},\;\text{for}\;\;t\geq 0, \end{array}$$

where *x*_1_*(*t*) is the periodic solution of the system

(32) $$\begin{array}{c} \frac{du}{dt}=u\left(t\right)\left(a_{10}-a_{11}u\left(t\right)\right),\;t\neq \left(n+l-1\right)T,\\ \Delta u\left(t\right)=-\alpha u\left(t\right),\;t=\left(n+l-1\right)T,\\ u\left(0^{+}\right)=x_{10}, \end{array}$$

*u*(*t*) → *x*_1_*(*t*), *t* → *∞*, and

(33) $$\begin{array}{c} \int_{0}^{T}x_{1}^{*}\left(t\right)dt=\frac{1}{a_{11}}\left[\ln\left(1-\alpha \right)+a_{10}T\right]. \end{array}$$

Similarly,

(34) $$\begin{array}{c} x_{2}\left(t\right)\leq x_{2}^{*}\left(t\right)+{ɛ}_{2},\;\text{for}\;\;t\geq 0, \end{array}$$

where *x*_2_*(*t*) is the periodic solution of the system

(35) $$\begin{array}{c} \frac{dv}{dt}=v\left(t\right)\left(a_{20}-a_{22}v\left(t\right)\right),\;t\neq nT,\\ \Delta v\left(t\right)=-\alpha v\left(t\right),\;t=nT,\\ v\left(0^{+}\right)=x_{20}, \end{array}$$

*v*(*t*) → *x*_2_*(*t*), *t* → *∞*, and

(36) $$\begin{array}{c} \int_{0}^{T}x_{2}^{*}\left(t\right)dt=\frac{1}{a_{22}}\left[\ln\left(1-\beta \right)+a_{20}T\right]. \end{array}$$

Therefore,

(37) $$\begin{array}{c} \frac{dx_{3}}{dt}\leq x_{3}\left(-a_{30}+a_{31}\left(x_{1}^{*}\left(t\right)+{ɛ}_{1}\right)+a_{32}\left(x_{2}^{*}\left(t\right)+{ɛ}_{2}\right)\right). \end{array}$$

Integrating (37) over ((*n* + *l* − 1)*T*, (*n* + *l*)*T*) yields

(38) x3((n+l)T)≤x3((n+l−1)T+) ×exp⁡∫(n+l−1)T(n+l)T(−a30+a31(x1∗(t)+ɛ1)+a32(x2∗(t)+ɛ2))dt≤x3((n+l−1)T) ×exp⁡(−a30T+a31a11(ln⁡⁡(1−α)+a10T)⁡+a32a22(ln⁡⁡(1−β)+a20T)+(a31ɛ1+a32ɛ2)T).=x3((n+l−1)T)ρ.

Therefore

(39) x3((n+l+k)T)≤x3((n+l+k−1)T)ρ≤x3((n+l−1)T)ρk.

*ρ* ^*k*^ → 0 as *k* → *∞*. This implies that *x*_3_(*t*) → 0 as *t* → *∞*.

Next, we prove that *x*_1_(*t*) → *x*_1_*(*t*) and *x*_2_(*t*) → *x*_2_*(*t*) as *t* → *∞* if lim⁡_*t*→*∞*_⁡*x*_3_(*t*) = 0. For *ɛ*_3_ > 0 there exists a *T*_1_ > 0 such that 0 < *x*_3_(*t*) < *ɛ*_3_, *t* > *T*_1_. Without loss of generality, we may assume that 0 < *x*_3_(*t*) < *ɛ*_3_ for all *t* ≥ 0. Then we have

(40) $$\begin{array}{c} \frac{dx_{1}}{dt}\geq x_{1}\left(a_{10}-a_{11}x_{1}-a_{13}{ɛ}_{3}\right),\;t\neq \left(n+l-1\right)T,\\ \Delta x_{1}\left(t\right)=-\alpha x_{1}\left(t\right),\;t=\left(n+l-1\right)T. \end{array}$$

By Lemmas 2 and 3, we have

(41) $$\begin{array}{c} x_{1}\left(t\right)\geq {\tilde{x}}_{1}\left(t\right)-{ɛ}_{1},\;\text{for}\;\;t\geq 0, \end{array}$$

where ${\tilde{x}}_{1}(t)$ is the periodic solution of the system

(42) $$\begin{array}{c} \frac{du}{dt}=u\left(t\right)\left(a_{10}-a_{11}u\left(t\right)-a_{13}{ɛ}_{3}\right),\;t\neq \left(n+l-1\right)T,\\ \Delta u\left(t\right)=-\alpha u\left(t\right),\;t=\left(n+l-1\right)T,\\ u\left(0^{+}\right)=x_{10}, \end{array}$$

$u(t)\to {\tilde{x}}_{1}(t)$ as *t* → *∞*, and

(43) $$\begin{array}{c} \int_{0}^{T}{\tilde{x}}_{1}\left(t\right)dt=\frac{1}{a_{11}}\left[\ln\left(1-\alpha \right)+\left(a_{10}-a_{13}{ɛ}_{3}\right)T\right]. \end{array}$$

Therefore, for *ɛ*_1_ > 0, we have

(44) $$\begin{array}{c} {\tilde{x}}_{1}\left(t\right)-{ɛ}_{1}\leq x_{1}\left(t\right)\leq x_{1}^{*}\left(t\right)+{ɛ}_{1} \end{array}$$

for *t* large enough. Let *ɛ*_3_ → 0, and we get *x*_1_*(*t*) − *ɛ*_1_ ≤ *x*_1_(*t*) ≤ *x*_1_*(*t*) + *ɛ*_1_ for large *t*, which implies *x*_1_(*t*) → *x*_1_*(*t*) as *t* → *∞*.

Similarly, we can get that *x*_2_(*t*) → *x*_2_*(*t*) as *t* → *∞*. This completes the proof.

**Remark**

Condition (20) can be rewritten as follows:

(45) $$\begin{array}{c} T<\frac{\left(a_{31}/a_{11}\right)\ln\left(1-\alpha \right)+\left(a_{32}/a_{22}\right)\ln\left(1-\beta \right)}{a_{30}-\left(a_{31}/a_{11}\right)a_{10}-\left(a_{32}/a_{22}\right)a_{20}}. \end{array}$$

Denote T* = ((*a*_31_/*a*_11_)ln⁡(1 − *α*) + (*a*_32_/*a*_22_)ln⁡(1 − *β*))/(*a*_30_ − (*a*_31_/*a*_11_)*a*_10_ − (*a*_32_/*a*_22_)*a*_20_), and we find that when *T* < T*, the giant panda-free periodic solution is globally asymptotically stable. That is to say, in this case, the giant panda will be extinct. In biology, when the period of bamboo flowing is smaller than the threshold T*, the bamboo cannot be revived to support giant panda again, so giant panda will die by starvation.

## 4. Permanence

We make mention of the definition of permanence before starting the permanence of system (1).

**Definition**

System (1) is said to be permanent if there exist two positive constants *m* and *M* such that every positive solution (*x*_1_(*t*), *x*_2_(*t*), *x*_3_(*t*)) of system (1) with *x*_10_, *x*_20_, *x*_30_ > 0 satisfies *m* ≤ *x*_1_(*t*) ≤ *M*, *m* ≤ *x*_2_(*t*) ≤ *M*, and *m* ≤ *x*_3_(*t*) ≤ *M* for sufficiently large *t*.

**Theorem**

Suppose that ln⁡(1 − *α*)+(*a*_10_ − *a*_13_ *M*)*T* > 0, ln⁡(1 − *β*)+(*a*_20_ − *a*_23_)*T* > 0, and

(46) −a30T+a31a11[ln⁡(1−α)+a10T]+a32a22[ln⁡(1−β)+a20T]  >0

hold, and system (1) is permanent, where *M* is upper bound of the solution of system (1).

**Proof**

Let (*x*_1_(*t*), *x*_2_(*t*), *x*_3_(*t*)) be a solution of (1). From Lemma 3, there exists a constant *M* > 0 such that *x*_1_(*t*) ≤ *M*, *x*_2_(*t*) ≤ *M*, *x*_3_(*t*) ≤ *M* for each solution *X* = (*x*_1_(*t*), *x*_2_(*t*), *x*_3_(*t*)) of (1) for all sufficiently large *t*. The first equation of (1) implies

(47) $$\begin{array}{c} x_{1}\left(t\right)\geq x_{1}\left(a_{10}-a_{11}x_{1}-a_{13}M\right). \end{array}$$

By Lemmas 2 and 3, we have

(48) $$\begin{array}{c} x_{1}\left(t\right)\geq u^{*}\left(t\right)-ɛ\equiv m_{1},\;\text{for}\;\;\text{sufficiently}\;\;\text{small}\;\;ɛ>0, \end{array}$$

where u*(*t*) is the periodic solution of system:

(49) $$\begin{array}{c} \frac{du}{dt}=u\left(t\right)\left(a_{10}-a_{11}u\left(t\right)-a_{13}M\right),\;t\neq \left(n+l-1\right)T,\\ \Delta u\left(t\right)=-\alpha u\left(t\right),\;t=\left(n+l-1\right)T,\\ u\left(0^{+}\right)=x_{10}, \end{array}$$

*u*(*t*) → u*(*t*), *t* → *∞*, and

(50) $$\begin{array}{c} \int_{0}^{T}u^{*}\left(t\right)dt=\frac{1}{a_{11}}\left[\ln\left(1-\alpha \right)+\left(a_{10}-a_{13}M\right)T\right]. \end{array}$$

Similarly, if ln⁡(1 − *β*) + (*a*_20_ − *a*_23_ *M*)*T* > 0 holds, we can get *x*_2_(*t*) > v*(*t*) − *ɛ* ≡ *m*_2_, where v*(*t*) is the periodic solution of system:

(51) $$\begin{array}{c} \frac{dv}{dt}=v\left(t\right)\left(a_{20}-a_{22}v\left(t\right)-a_{23}M\right),\;t\neq nT,\\ \Delta v\left(t\right)=-\alpha v\left(t\right),\;t=nT,\\ v\left(0^{+}\right)=x_{10}, \end{array}$$

*v*(*t*) → v*(*t*), *t* → *∞*, and

(52) $$\begin{array}{c} \int_{0}^{T}v^{*}\left(t\right)dt=\frac{1}{a_{22}}\left[\ln\left(1-\alpha \right)+\left(a_{20}-a_{23}M\right)T\right]. \end{array}$$

Therefore, it is necessary only to find an *m*_3_ > 0 such that *x*_3_(*t*) ≥ *m*_3_ for sufficiently large *t*. This can be done in the following two steps.

*Step 1*. Choose *m*_0_ > 0, *ɛ*_4_ > 0, *ɛ*_5_ > 0 small enough that if condition (46) holds,

(53) σ=exp⁡⁡(−a30T−a33m0T−a31ɛ4T−a32ɛ5T+a31a11[ln⁡(1−α)+(a10−a13m0)T])+a32a22[ln⁡(1−β)+(a20−a23m0)T])>1.

This step will show that *x*_3_(*t*) ≥ *m*_0_ for some *t*_1_ > 0. Assuming the contrary, *x*_3_(*t*) < 0 for all *t*, from system (1), we have

(54) $$\begin{array}{c} \frac{dx_{1}}{dt}\geq x_{1}\left(a_{10}-a_{11}x_{1}-a_{13}m_{0}\right),\;t\neq \left(n+l-1\right)T,\\ \frac{dx_{2}}{dt}\geq x_{2}\left(a_{20}-a_{22}x_{2}-a_{23}m_{0}\right),\;t\neq nT,\\ \frac{dx_{3}}{dt}\geq x_{3}\left(-a_{30}-a_{33}m_{0}+a_{31}x_{1}+a_{32}x_{2}\right),\\ \Delta x_{1}\left(t\right)=-\alpha x_{1}\left(t\right),\;t=\left(n+l-1\right)T,\\ \Delta x_{2}\left(t\right)=-\beta x_{2}\left(t\right),\;t=nT,\\ \left(x_{1}\left(0^{+}\right),x_{2}\left(0^{+}\right),x_{3}\left(0^{+}\right)\right)=\left(x_{10},x_{20}x_{30}\right). \end{array}$$

Consider the corresponding impulsive compare system:

(55) $$\begin{array}{c} \frac{du\left(t\right)}{dt}=u\left(t\right)\left(a_{10}-a_{11}u\left(t\right)-a_{13}m_{0}\right),\;t\neq \left(n+l-1\right)T,\\ \frac{dv\left(t\right)}{dt}=v\left(t\right)\left(a_{20}-a_{22}v\left(t\right)-a_{23}m_{0}\right),\;t\neq nT,\\ \frac{dw}{dt}=w\left(t\right)\left(-a_{30}-a_{33}m_{0}+a_{31}u\left(t\right)+a_{32}v\left(t\right)\right),\\ \Delta u\left(t\right)=-\alpha u\left(t\right),\;t=\left(n+l-1\right)T,\\ \Delta v\left(t\right)=-\beta v\left(t\right),\;t=nT,\\ \left(u\left(0^{+}\right),v\left(0^{+}\right),w\left(0^{+}\right)\right)=\left(x_{10},x_{20},x_{30}\right). \end{array}$$

By Lemma 2, *x*_1_(*t*) ≥ *u*(*t*), *x*_2_(*t*) > *v*(*t*), and *x*_3_(*t*) ≥ *w*(*t*). By Lemma 3,

(56) $$\begin{array}{c} u^{*}\left(t\right)-{ɛ}_{4}\leq u\left(t\right)\leq u^{*}\left(t\right)+{ɛ}_{4},\;\text{for}\;\;\text{sufficiently}\;\;\text{small}\;\;{ɛ}_{4},\\ \int_{0}^{T}u^{*}\left(t\right)dt=\frac{1}{a_{11}}\left[\ln\left(1-\alpha \right)+\left(a_{10}-a_{13}m_{0}\right)T\right]. \end{array}$$

Also,

(57) $$\begin{array}{c} v^{*}\left(t\right)-{ɛ}_{5}\leq v\left(t\right)\leq v^{*}\left(t\right)+{ɛ}_{5},\;\text{for}\;\;\text{sufficiently}\;\;\text{small}\;\;{ɛ}_{2},\\ \int_{0}^{T}v^{*}\left(t\right)dt=\frac{1}{a_{22}}\left[\ln\left(1-\beta \right)+\left(a_{20}-a_{23}m_{0}\right)T\right]. \end{array}$$

Thus

(58) dwdt≥(−a30−a30m0+a31(u∗(t)−ɛ4)+a32(v∗(t)−ɛ5))w(t).

Integrating (58) over ((*n* + *l* − 1)*T*, (*n* + *l*)*T*) yields

(59) w((n+l)T)≥w((n+l−1)T+) ×exp⁡∫(n+l−1)T(n+l)T(−a30−a33m0+a31(u∗(t)−ɛ4)+a32(v∗(t)−ɛ5))dt≥w((n+l−1)T)σ.

Therefore

(60) x3((n+l+k)T)≥w((n+l+k)T)≥w((n+l+k−1)T)σ≥w((n+l−1)T)σk.

Since *σ* > 1, *σ* ^*k*^ → *∞* as *k* → *∞*. This implies that *x*_3_(*t*) → *∞* as *t* → *∞*, which contradicts the boundedness of *x*_3_(*t*).

*Step  2*. If *x*_3_(*t*) ≥ *m*_0_ for all *t* ≥ *t*_1_, then the proof is complete. If not, let t* = inf⁡_*t*≥*t*_1__⁡{*x*_3_(*t*) < *m*_0_}, and then *x*_3_(*t*) ≥ *m*_0_ for *t* ∈ [*t*_1_, t*) and *x*_3_(t*) = *m*_0_. By Step 1, there exists a *t*′ > t* such that *x*(*t*′) ≥ *m*_0_. Set *t*_2_ = inf⁡_*t*>t*_⁡{*x*_3_(*t*) ≥ *m*_0_}, and then *x*_3_(*t*) < *m*_0_ for *t* ∈ (t*, *t*_2_) and *x*_3_(*t*_2_) = *m*_0_. This process can be continued by repeating Step 1. If this process steps after a finite number of repetitions, the proof is complete. If not, there exists an interval sequence [*t*_*n*_, *t*_*n*+1_], (*n* ∈ *ℕ*), such that *x*_3_(*t*) ≥ *m*_0_, *t* ∈ [*t*_*n*_, *t*_*n*+1_]. Let *T*′ = sup⁡|*t*_*n*+1_ − *t*_*n*_|, *n* ∈ *ℕ*. If *T*′ = *∞*, there must exist a subsequence *t*_*n*_*i*__ such that *t*_*n*_*i*_+1_ − *t*_*n*_*i*__ → *∞* as *n*_*i*_ → *∞*. From Step 1, this can lead to a contradiction with the boundedness of *x*_3_(*t*); therefore, *T*′ < *∞*. Then

(61) x3(t)≥x3(tn) ×exp⁡∫tnt(−a30−a33M+a31u∗(t)     −a31ɛ4+a32v∗(t)−a32ɛ5)ds≥m0exp⁡((−a30−a33M)T′)≡m3.

Let *m* = min⁡{*m*_1_, *m*_2_, *m*_3_}, and then

(62) $$\begin{array}{c} \underset{t\to \infty }{\mathrm{liminf}}x_{1}\left(t\right)\geq m,\;\;\underset{t\to \infty }{\mathrm{liminf}}x_{2}\left(t\right)\geq m,\\ \underset{t\to \infty }{\mathrm{liminf}}x_{3}\left(t\right)\geq m. \end{array}$$

This completes the proof.

Next, we consider the following two subsystems:

(63) dx1dt=x1(a10−a11x1−a13x3), t≠(n+l−1)T,dx3dt=x3(a30−a33x3+a31x1),Δx1(t)=−αx1(t), t=(n+l−1)T,(x1(0+),x3(0+))=(x10,x30),

(64) dx2dt=x2(a20−a22x2−a23x3), t≠nT,dx3dt=x3(a30−a33x3+a32x2),Δx2(t)=−βx2(t), t=nT,(x2(0+),x3(0+))=(x20,x30).

Imitating the proof of Theorem 12, we can obtain the following theorems.

**Theorem**

Subsystem (63) is permanent if

(65) $$\begin{array}{c} -a_{30}T+\frac{a_{31}}{a_{11}}\left[\ln\left(1-\alpha \right)+a_{10}T\right]>0 \end{array}$$

holds.

**Theorem**

Subsystem (64) is permanent if

(66) $$\begin{array}{c} -a_{30}T+\frac{a_{31}}{a_{22}}\left[\ln\left(1-\beta \right)+a_{20}T\right]>0 \end{array}$$

holds.

**Remark**

In this paper, our purpose is that of considering the survival of the giant panda. From Theorems 13 and 14, we can easily obtain that either condition (65) or condition (66) holds, and the giant panda can be persistent. Clearly, this condition is weaker than condition (46). Moreover, it is also weaker than that of only one kind of food bamboo in the habitat of giant panda.

## 5. Conclusion

In this paper, we consider an impulsive differential system of the population ecology on the three populations of the giant panda and two kinds of bamboo. The local and global stability of the giant panda-free periodic solution are obtained and we find the threshold value T*. When *T* < T*, the giant panda-free periodic solution is globally asymptotically stable. That is to say, the giant panda will be extinct if the period of bamboo flowering is smaller than the threshold T*, because the bamboo cannot be revived to support giant panda again. Comparing Theorem 12 with Theorems 13 and 14, we know that when there are two kinds of staple bamboo in the giant panda habitat, the conditions which guarantee the giant panda to be permanent are weaker than that of only one kind of staple bamboo in the habitat. Our results will provide a theoretical basis for the rejuvenation update of the bamboo forest after bamboo flowering. Once the bamboo forest flowers, we should timely remove flowering bamboo stains or clamps, and we can promote the flowering bamboo to update and restore as soon as possible by manual intervention approach, such as excavating bamboo stump and rhizome of flowering bamboo, loosing soil and fertilizing Nitrogen fertilizer in the whole forest to promote new whip growth, and sprouting bamboo into bamboo. Our results also provide a theoretical basis for the implementation of artificial bamboo forest. We can select giant panda staple bamboo species according to the flowering cycle to implement artificial bamboo forest plan.
